# Comprehensive Analysis of a Competing Endogenous RNA Network Identifies Seven-lncRNA Signature as a Prognostic Biomarker for Melanoma

**DOI:** 10.3389/fonc.2019.00935

**Published:** 2019-10-03

**Authors:** Nian Liu, Zijian Liu, Xinxin Liu, Hongxiang Chen

**Affiliations:** ^1^Department of Dermatology, Union Hospital, Tongji Medical College, Huazhong University of Science and Technology, Wuhan, China; ^2^Cancer Center, Tongji Medical College, Union Hospital, Huazhong University of Science and Technology, Wuhan, China

**Keywords:** melanoma, lncRNA, ceRNA, prognostic, MIR205HG, MIAT

## Abstract

Long non-coding RNAs (LncRNAs) can act as competing endogenous RNA (ceRNA) involving in tumor initiation and progression. Nevertheless, the prognostic roles of lncRNAs in lncRNA-related ceRNA network of melanoma remain elusive. In this study, RNA sequence profiles were downloaded from The Cancer Genome Atlas (TCGA) database, and there were 2020 differentially expressed messenger RNAs (DEmRNAs), 438 differentially expressed lncRNAs (DElncRNAs) and 65 differentially expressed microRNAs (DEmiRNAs) between primary and metastasis melanoma patients. A ceRNA regulatory network was constructed based on the DElncRNAs-DEmiRNAs and DEmiRNAs-DEmRNAs interactions, which contained 39 lncRNAs, 10 miRNAs, and 16 mRNAs. Furthermore, univariate and multivariate Cox regression analysis were carried out to establish a 7-lncRNA prognostic signature. Subsequently, the area under the curve (AUC) value of the receiver operating characteristic (ROC) curve and the Kaplan-Meier risk survival analysis revealed the significant performance of this signature. Finally, pathway enrichment analyses implied that lncRNA MIR205HG and MIAT were associated with multiple cancer-related pathways, especially epidermis development and immune response. The current study provides novel insights into the lncRNA-related ceRNA network and the potential of lncRNAs to be candidate prognostic biomarkers and therapeutic targets in melanoma.

## Introduction

Melanoma, known as a highly aggressive skin tumor, arises through malignant transformation of melanocytes and is the leading cause of skin cancer-related deaths (1). According to the global cancer statistics carried in 2018, melanoma is on the increase with around 287,723 new cases and about 60,712 cancer deaths at an estimate (2). Despite several studies have showed surgical excision of primary melanoma (PM) with high cure rate, metastatic melanoma (MM) presents a life-threatening behavior (3). To date, a lot of work has been done in melanoma, but the underlying molecular mechanisms still remain unclear (4, 5). Therefore, it is vital to identify potential molecular diagnostic markers and therapeutic targets in melanoma, particularly the metastatic form.

Long non-coding RNAs (LncRNAs) are defined as any ncRNA that is longer than 200 nucleotides in length, which was once regarded as the “dark matter” for the limited or no protein-coding capacity (6). It has been reported that lncRNAs possess the capacity to regulate microRNAs' (miRNAs) function through miRNA sponge effect (6). This miRNA-regulated lncRNA mechanism is a part of the “competing endogenous RNA (ceRNA) hypothesis,” which was firstly proposed by Salmena et al. (7). In the ceRNA regulatory network, lncRNAs can act as endogenous molecular sponges to regulate the expression of messenger RNAs (mRNAs) through miRNA response elements (8). A growing number of researches have confirmed that lncRNAs are involved in the occurrence and progress of cancers through a disrupt in the balance of ceRNA networks, like prostate and colorectal cancer (9, 10).

In the past decades, several studies have investigated the potential role of lncRNAs as diagnostic or prognostic targets in cancers, such as breast cancer (11), gastric cancer (12), lung cancer (13), and melanoma (14–16). Fifteen lncRNAs were significantly correlated with the prognosis of patients with melanoma in an integrated bioinformatics analysis (14). Moreover, Chen et al. (15) and Yang et al. (16) suggested a 4-lncRNA (HCP5, LIMD1-AS1, MIR155HG, and UNQ6494) and a 6-lncRNA (AL050303, LINC00707, LINC01324, RP11-85G21, RP4-794I6.4, and RP5-855F16) signature for predicting the prognosis of melanoma patients, respectively. However, the association of lncRNAs with the prognosis of melanoma patients still remains elusive.

In the present study, the Cancer Genome Atlas (TCGA) database was used to obtain gene expression profiles to identify differentially expressed (DE) mRNAs, lncRNAs, and miRNAs between in PM and MM. Based on the above DERNAs, the lncRNA-miRNA-mRNA ceRNA network was established. Subsequently, we identified 7-lncRNA model with prognostic value based on the ceRNA regulatory network and these prognostic lncRNAs have been well-explored for biological function in melanoma. This study aimed to identify skin cancer-specific lncRNAs that are involving in prognosis and progression in melanoma.

## Materials and Methods

### Patient Information and Data Processing

By virtue of genomic data commons data portal provided officially by TCGA database (https://cancergenome.nih.gov/) (17), clinical information and transcriptome profiles containing mRNA, lncRNA, and miRNA, were downloaded. A total of 435 melanoma samples were included in our analysis, comprising 96 PM samples and 339 MM samples. This study strictly adhere to the publication guidelines provided by TCGA (https://cancergenome.nih.gov/ publications/publicationguidelines) and as our data was obtained from TCGA database (version 2019/03/26 v16.0), there was no requirement for ethics committee approval.

### Analysis of Differential Expression Profiles

The RNA expression data of lncRNAs, miRNAs, and mRNAs from TCGA database were processed. DERNAs between PM and MM were analyzed using the edge R package to identify potential molecules during the development of primary and metastatic status of melanoma. And |log_2_FC| >1 and false discovery rate (FDR) <0.05 were used as cut-off criteria.

### Construction of CeRNA Regulatory Network

Based on base complementary pairing principle and experimental verification, some authoritative databases provided a relationship pair such as lncRNA-miRNA and miRNA-mRNA, which can be effective for us to construct a ceRNA network centered on miRNA. Firstly, we used the miRcode database (http://www.mircode.org/) (18) to predict lncRNA-miRNA interactions, which were then combined with selected miRNAs. Secondly, three databases including Targetscan (http://www.targetscan.org/) (19), miRTarBase (http://mirtarbase.mbc.nctu.edu.tw/) (20), miRDB (http://www.mirdb.org/) (21) were resorted to predict corresponding target mRNAs of miRNAs, respectively. Finally, miRNAs that interacted with both lncRNAs and mRNAs were selected to construct the ceRNA regulatory network using Cytoscape 3.6.1 visualization software (https://cytoscape.org/) (22).

### Construction of Melanoma-Specific Prognostic Model

Univariate Cox proportional hazards regression analyses were performed based on melanoma-specific DElncRNAs in the ceRNA network. The lncRNA with a *P*-value of <0.05 was included in the subsequent analysis. Thereafter, we constructed a prognostic predictive model and obtained a combined prognosis score system based on those DElncRNAs. The risk score was computed as follows: Risk score = ∑βi×expRNAi, where expRNA is the expression level of RNA and β is the regression coefficient derived from the multivariate Cox regression model (23).

According to this risk score formula and our identified lncRNA signature, the patients with melanoma were classified into a high- or low-risk group based on a median risk score cut-off value. Differences of overall survival (OS) were exited between the two groups. *P* < 0.05 was regarded as significant unless specifically indicated. The “survival ROC” package in R was used to construct the time-dependent receiver operating characteristic (ROC) curves within 3, 5, and 10 years as the defining point, and assess the sensitivity and specificity of the lncRNAs signatures in predicting survival. *P* < 0.05 was considered statistically significant. Kaplan–Meier analysis was used to determine the overall survival rate between groups. All of these analyses were performed in R software (version 3.5.2) (24).

### Function Enrichment Analysis

Gene ontology (GO) and Kyoto Encyclopedia of Genes and Genomes (KEGG) functional enrichment analyses were performed to predict the biological function of prognostic lncRNAs using Database for Annotation, Visualization and Integrated Discovery (DAVID) (25), a high-throughput and integrated data-mining environment. Enrichment analysis was carried out using the functional annotation chart and functional annotation clustering options, containing KEGG pathways and GO terms. *P* < 0.05 was considered to indicate statistical significance.

The microenvironment cell populations-counter (MCP-counter) was used to evaluate the association between genes and immune cell populations, using genometric mean of expression of the transcriptomic markers.

### Statistical Analysis

All of the statistical analyses were performed in R software (version 3.5.2). *P* < 0.05 was considered statistically significant unless otherwise mentioned.

## Results

### Identification of DERNAs in Melanoma

A total of 435 skin cutaneous melanoma samples were obtained from TCGA database after data preprocessing. Based on the screening criteria of |log_2_FC| >1 and FDR <0.05, 2,020 DEmRNAs (1,369 up-regulated and 651 down-regulated), 438 DElncRNAs (348 up-regulated and 90 down-regulated) and 65 DEmiRNAs (48 up-regulated and 17 down-regulated) were identified between PM and MM. Among them, top 10 DEmRNAs, DElncRNAs, and DEmiRNAs were shown in Table 1.

**Table 1:** Top 10 up-regulated and down-regulated differentially expressed mRNAs, lncRNAs, and miRNAs between PM and MM.

**mRNA**	**Log_**2**_FC**	***P*-value**	**FDR**	**lncRNA**	**Log_**2**_FC**	***P*-value**	**FDR**	**miRNA**	**Log_**2**_FC**	***P-*value**	**FDR**
CYP11B1	9.50	2.81E-15	9.77E-14	LINC00200	6.87	4.86E-16	1.83E-14	hsa-mir-521-1	2.97	4.24E-05	0.000324746
OTOR	9.41	2.68E-13	7.56E-12	LINC00824	5.60	3.36E-19	1.65E-17	hsa-mir-599	2.89	1.24E-05	0.000123639
SFTPA2	8.75	3.32E-19	1.64E-17	AC090150.1	5.25	2.15E-08	2.69E-07	hsa-mir-1323	2.31	0.000164931	0.00097977
SFTPB	8.43	1.14E-25	8.00E-24	AC112176.1	5.02	4.55E-09	6.50E-08	hsa-mir-3681	2.31	9.69E-08	1.86E-06
SCGB1A1	8.27	9.99E-17	4.06E-15	AC104051.2	4.75	1.50E-12	3.85E-11	hsa-mir-520a	2.18	0.000115439	0.000738515
SFTPA1	7.65	3.69E-12	8.98E-11	AC104823.1	4.74	3.07E-11	6.45E-10	hsa-mir-527	2.12	0.001674285	0.006377622
STATH	7.50	1.95E-11	4.24E-10	LINC00967	4.64	1.44E-09	2.29E-08	hsa-mir-153-2	2.10	2.94E-14	1.13E-12
SCGB3A2	7.25	3.38E-15	1.16E-13	LINC00207	4.63	5.27E-08	6.09E-07	hsa-mir-372	2.04	9.19E-06	9.97E-05
CYP17A1	7.07	2.60E-26	1.88E-24	LINC01425	4.35	1.18E-09	1.90E-08	hsa-mir-675	2.01	1.79E-16	8.12E-15
CSN2	6.98	1.99E-11	4.30E-10	AC083967.1	4.33	7.64E-22	4.35E-20	hsa-mir-519c	1.91	0.003929176	0.012178004
C10orf99	−6.97	5.50E-53	1.28E-50	AL049555.1	−3.70	2.90E-27	2.19E-25	hsa-mir-200a	−2.34	8.74E-32	7.27E-30
PI3	−7.09	1.63E-102	2.72E-99	AC245041.1	−4.13	3.23E-24	2.11E-22	hsa-mir-200b	−2.44	2.48E-31	1.77E-29
S100A7	−7.12	5.54E-62	1.90E-59	AL512274.1	−4.22	1.88E-87	2.04E-84	hsa-mir-141	−2.61	3.70E-32	3.69E-30
KRT6C	−7.18	9.64E-51	1.95E-48	C7orf71	−4.28	1.19E-47	2.12E-45	hsa-mir-203b	−2.67	7.28E-15	3.03E-13
LCE3E	−7.53	8.37E-51	1.73E-48	FAM83A-AS1	−4.55	3.66E-58	1.06E-55	hsa-mir-200c	−2.89	1.80E-39	2.24E-37
S100A7A	−7.88	4.78E-58	1.36E-55	LINC02560	−4.83	5.90E-69	2.90E-66	hsa-mir-944	−3.28	1.00E-46	2.50E-44
WFDC12	−7.90	2.74E-75	1.98E-72	MIR205HG	−4.85	7.71E-32	7.13E-30	hsa-mir-6510	−3.63	1.68E-16	8.12E-15
SPRR2G	−8.24	6.73E-69	3.24E-66	FAM41C	−5.42	4.83E-61	1.59E-58	hsa-mir-203a	−4.00	2.57E-40	4.28E-38
DEFB4A	−8.29	1.54E-77	1.19E-74	AC022081.1	−5.59	2.76E-36	3.20E-34	hsa-mir-205	−4.37	4.54E-29	2.83E-27
KRT9	−9.57	9.10E-93	1.09E-89	LINC01527	−6.35	9.82E-44	1.51E-41	hsa-mir-122	−5.41	6.70E-49	3.35E-46

### Construction of CeRNA Regulatory Network

To explore the targeting relationship of the DERNAs, we focused on the interaction of miRNAs with lncRNAs and mRNAs. Firstly, we explored the regulatory loops with lncRNA-miRNA in the miRcode database, and found that 39 of 438 specific DElncRNAs might target 10 of 65 specific DEmiRNAs (Table 2). Subsequently, MiRTarBase, Targetscan, and miRDB database were used to identify miRNA-targeted mRNAs, involving 9 miRNAs and 16 mRNAs (Table 3).

**Table 2:** DElncRNAs-targeted melanoma-specific intersection miRNAs.

**lncRNA**	**miRNA**
MEG3	hsa-mir-372, hsa-mir-373, hsa-mir-141, hsa-mir-200a, hsa-mir-150, hsa-mir-206, hsa-mir-205, hsa-mir-217, hsa-mir-122
AGAP11	hsa-mir-372, hsa-mir-373, hsa-mir-141, hsa-mir-200a, hsa-mir-150, hsa-mir-206, hsa-mir-205, hsa-mir-217
MIAT	hsa-mir-372, hsa-mir-373, hsa-mir-141, hsa-mir-200a, hsa-mir-150, hsa-mir-206, hsa-mir-205
ADAMTS9-AS2	hsa-mir-372, hsa-mir-373, hsa-mir-141, hsa-mir-200a, hsa-mir-150, hsa-mir-205, hsa-mir-122
SOX2-OT	hsa-mir-141, hsa-mir-200a, hsa-mir-206, hsa-mir-205, hsa-mir-122
MDS2	hsa-mir-141, hsa-mir-200a, hsa-mir-150, hsa-mir-205, hsa-mir-122
KIAA0087	hsa-mir-141, hsa-mir-200a, hsa-mir-150, hsa-mir-217
C17orf77	hsa-mir-372, hsa-mir-373, has-mir-150, has-mir-206
FAM41C	hsa-mir-141, hsa-mir-200a, hsa-mir-206, hsa-mir-205
MIR205HG	hsa-mir-150, hsa-mir-206, hsa-mir-205, hsa-mir-122
NALCN-AS1	hsa-mir-372, hsa-mir-373, hsa-mir-150, hsa-mir-205
MYCNOS	hsa-mir-150, hsa-mir-205, hsa-mir-217, hsa-mir-122
LINC00402	hsa-mir-141, hsa-mir-200a, hsa-mir-150, hsa-mir-217
LIFR-AS1	hsa-mir-372, hsa-mir-373, hsa-mir-150, hsa-mir-206
LINC00322	hsa-mir-372, hsa-mir-373, hsa-mir-150
C10orf91	hsa-mir-372, hsa-mir-373, hsa-mir-122
MIR137HG	hsa-mir-100, hsa-mir-150, hsa-mir-217
PLCH1-AS1	hsa-mir-372, hsa-mir-373, hsa-mir-217
ZBTB20-AS1	hsa-mir-206, hsa-mir-205, hsa-mir-217
H19	hsa-mir-141, hsa-mir-200a, hsa-mir-206
IGF2-AS	hsa-mir-150, hsa-mir-122
AC108134.1	hsa-mir-150, hsa-mir-206
LINC00393	hsa-mir-372, hsa-mir-373
LINC00200	hsa-mir-150, hsa-mir-217
LINC00326	hsa-mir-150, hsa-mir-205
LINC00460	hsa-mir-150, hsa-mir-206
AC011374.1	hsa-mir-372, hsa-mir-373
AC110619.1	hsa-mir-206, hsa-mir-122
LINC00266-1	hsa-mir-217
LINC00207	hsa-mir-206
AL163952.1	hsa-mir-205
C7orf71	hsa-mir-217
AL161431.1	hsa-mir-150
KIF25-AS1	hsa-mir-150
DNM3OS	hsa-mir-150
AC024597.1	hsa-mir-122
ADAMTS9-AS1	hsa-mir-150
AP000662.1	hsa-mir-122
AC012640.1	hsa-mir-205

**Table 3:** DEmiRNAs-targeted melanoma-specific intersection mRNAs.

**miRNA**	**mRNA**
hsa-mir-141	STAT4, EPHA7, ELAVL2, ZEB1, PTPRD
hsa-mir-200a	PTPRD, ELAVL2, ZEB1, EPHA7
hsa-mir-372	MIXL1, SIK1, TMEM100, ELAVL2
hsa-mir-373	ELAVL2, MIXL1, SIK1, TMEM100
hsa-mir-205	ESRRG, ZEB1, ENPP4
hsa-mir-150	ZEB1, RNF165, MUC4
hsa-mir-206	SFRP1, GJA1
hsa-mir-217	GPC5
hsa-mir-100	KBTBD8

**Figure 1:**
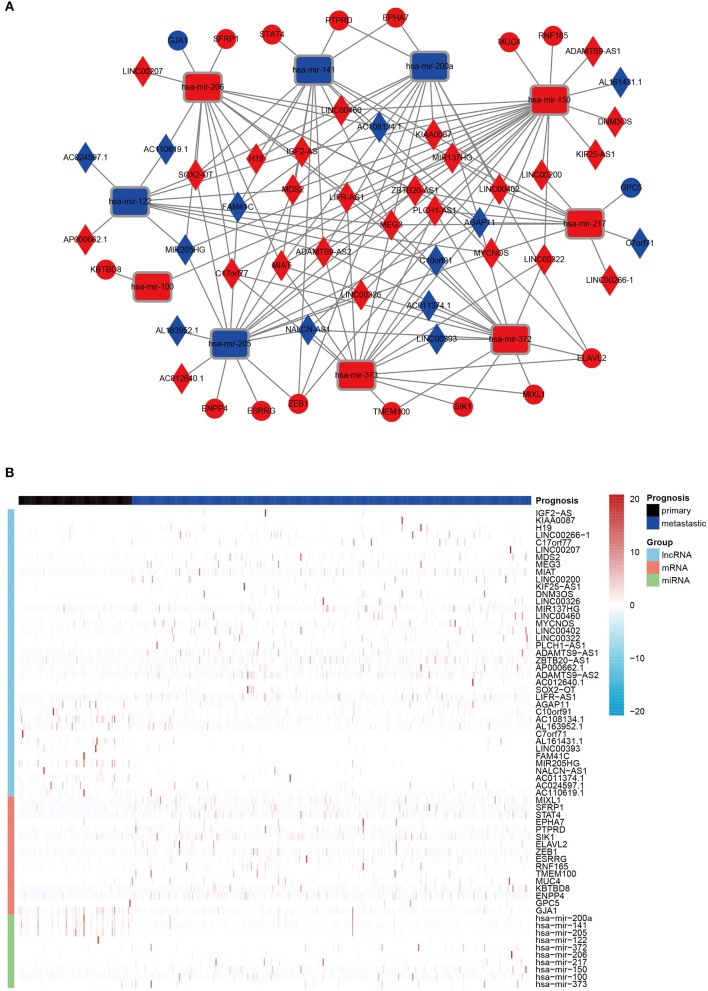
The DERNAs in ceRNA network. **(A)** A global view of the ceRNA regulatory network in melanoma. Rectangles, miRNAs; rhombuses, lncRNAs; circles, mRNAs; red, up-regulation; blue, down-regulation. **(B)** A heatmap of DE lncRNAs, miRNAs, and mRNAs in the ceRNA network.

Finally, on account of the regulatory pairs of lncRNA-miRNA and miRNA-mRNA, a lncRNA-miRNA-mRNA ceRNA network was constructed, using Cytoscape 3.6.1 software. In total, 39 lncRNAs, 10 miRNAs, and 16 mRNAs were included in the ceRNA regulatory network, containing 65 nodes and 145 edges (Figure 1A). Furthermore, the dysregulation of the members in the ceRNA network was displayed in a heatmap (Figure 1B).

### Identification of a Seven-LncRNA Prognostic Signature

**Figure 2:**
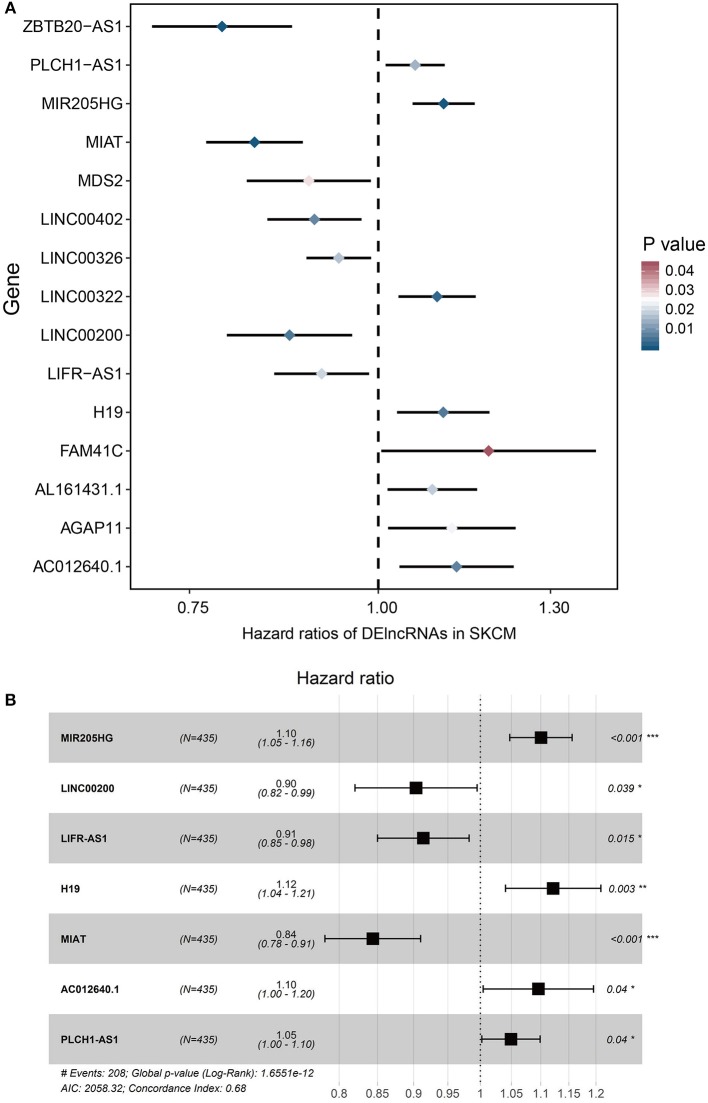
Cox regression analyses of DE lncRNAs based on the ceRNA network. **(A)** 15 DElncRNAs that were significantly correlated with overall survival derived from the univariable Cox regression analysis in melanoma patients. **(B)** 7 DElncRNAs that were significantly correlated with overall survival derived from the multivariate Cox regression analysis in melanoma patients.

To determine the association between lncRNAs and patients' outcomes, each DElncRNA in the ceRNA network was firstly submitted to univariate Cox proportional hazards regression. Fifteen DElncRNAs in the ceRNA network were identified to have a significant prognostic value (*P* < 0.05; Figure 2A). Then, a predictive model was constructed based on the coefficient of the 7 lncRNAs obtained from multivariate Cox proportional hazards regression analysis: MIR205HG, LINC00200, LIFR-AS1, H19, MIAT, AC012640.1, and PLCH1-AS1 (*P* < 0.05; Supplementary Table 1, Figure 2B and Table 4). The risk-score formula used to calculate the risk score was as follows: (0.0957^*^MIR205HG) + (−0.1016^*^LINC00200) + (−0.0902^*^LIFR-AS1) + (0.1149^*^H19) + (−0.169^*^MIAT) + (0.0915^*^AC012640.1) + (0.0483^*^PLCH1-AS1). As shown in Figure 2B, the Concordance index of this prognostic model was 0.68.

**Table 4:** Multivariate Cox regression analysis of 7 prognostic lncRNAs associated with overall survival in melanoma patients.

**lncRNA**	**Coef**	**Exp(coef)**	**Se(coef)**	**z**	***P***
MIR205HG	0.096	1.100	0.025	3.81	0.00014
LINC00200	−0.102	0.903	0.049	−2.06	0.03904
LIFR-AS1	−0.090	0.914	0.037	−2.44	0.01455
H19	0.115	1.122	0.038	2.99	0.00278
MIAT	−0.170	0.844	0.039	−4.40	1.07E-05
AC012640.1	0.091	1.096	0.044	2.06	0.03975
PLCH1-AS1	0.048	1.050	0.024	2.06	0.03980

### Risk Stratification and ROC Curve Analysis

**Figure 3:**
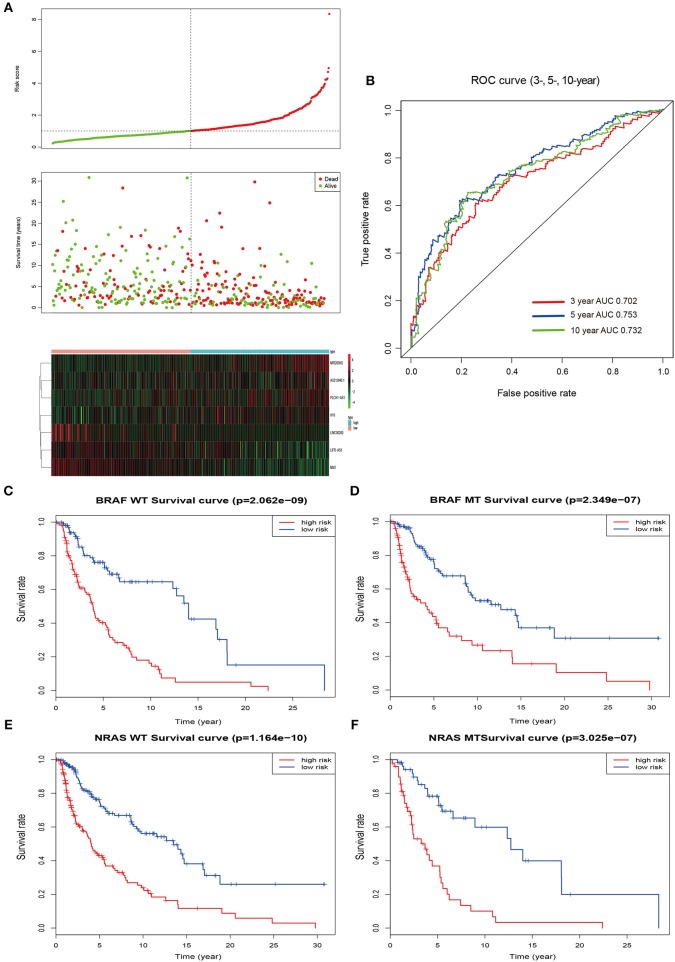
Risk score by the 7-lncRNA signature, time-dependent ROC curve and Kaplan–Meier survival analysis. **(A)** The distribution of the risk score, survival status, and 7-lncRNA signature. **(B)** ROC curve was plotted for 3-, 5-, and 10-year overall survival in melanoma. red, 3-year; blue, 5-year; green, 10-year. **(C,D)** Kaplan–Meier estimates of the overall survival of patients carrying wild-type and mutant BRAF. **(E,F)** Kaplan–Meier analysis estimates the overall survival of patients carrying wild-type and mutant NRAS. WT, wild type; MT, mutation; red, high risk; blue, low risk.

According to the median value of the prognostic risk score, patients were divided into low- and high-risk groups. The distribution of the risk score along with the corresponding overall survival data and the expression level of 7 lncRNAs in the prognostic model were plotted and shown in Figure 3. As depicted in the picture, patients with higher risk scores tended to experience a shorter overall survival time and higher death rate (*P* < 0.001; Figure 3A). Additionally, we used time-dependent ROC analysis to assess the prognostic significance of 7-lncRNA. The area under the curve (AUC) value of ROC analysis for the prognostic signature was 0.702, 0.753, and 0.732 for 3-year survival, 5-year survival, and 10-year survival, respectively (Figure 3B).

Mutations of the oncogenes BRAF and NRAS are the most common genetic alterations in cutaneous melanoma and the prognostic significance of the mutations shows differently (26). In order to better evaluate the accuracy of the model, we checked the effect of BRAF and NRAS mutation or wild-type on the prognostic ability of melanoma in the same cohort. Kaplan–Meier analysis showed that the higher risk score was associated with a higher mortality risk in the wild type or mutant type of these two genes (Figures 3C–F).

### Functional Characteristics of Prognostic LncRNAs

**Figure 4:**
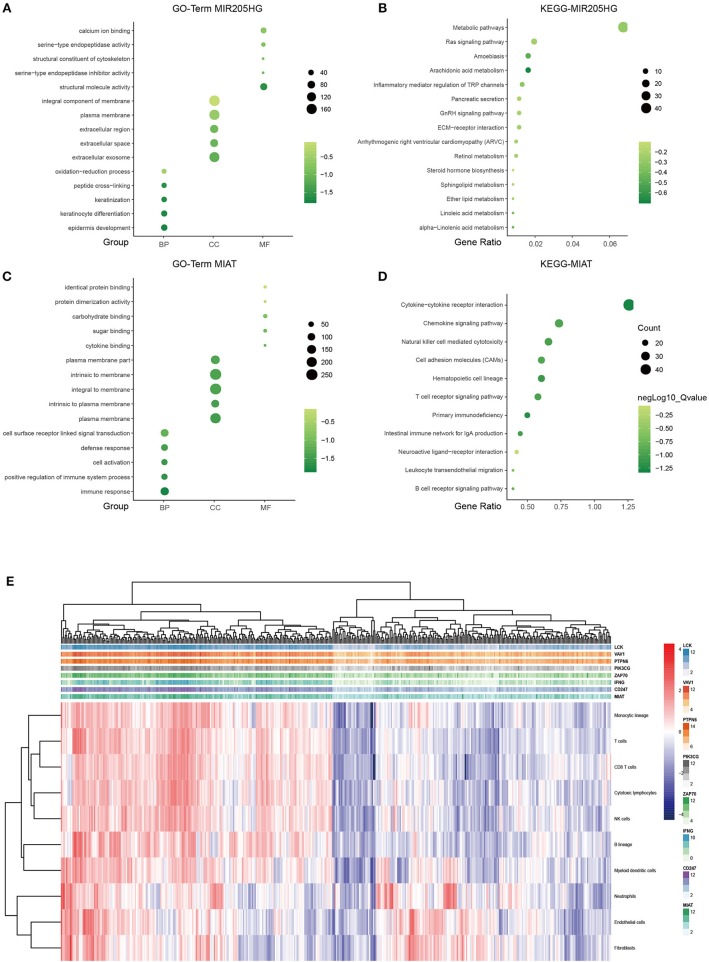
LncRNA MIR205HG and MIAT function prediction. GO functional enrichment analysis of MIR205HG **(A)** and MIAT **(C)**. X axis, GO term group; Y axis, GO terms. KEGG pathway enrichment analysis of MIR205HG **(B)** and MIAT **(D)**. X axis, gene ratio; Y axis, KEGG pathway terms. light green, low enrichment; dark green, high enrichment. **(E)** Association between MIAT-mediated immune molecules expression and immune cell populations in melanoma. X axis: genes; Y axis: MIAT, MIAT-related immune molecules and immune cell. Red: high enrichment; blue: low enrichment. BP, biological process; CC, cellular component; MF, molecular function.

It is well-known that coexpression genes pairs are more likely to be functionally related (27). To elucidate the underlying biological mechanism of the prognostic 7-lncRNA, we applied Pearson correlation coefficient to calculate the coexpression relationship between the expression levels of the 7 lncRNAs and mRNAs. In order to make the function prediction more reliable, we intersected the up- and down- regulated DEmRNAs with the positively or negatively correlated coding genes, respectively. The inclusion criteria of coexpressed gene were as follows: (a) Person correlation coefficient >0.4, and (b) identified from DEmRNAs mentioned above. The results showed that only MIR205HG and MIAT could meet the cut-off value, possibly because of other components with low potential biological functions (Supplementary Figure 1). Therefore, MIR205HG and MIAT were regarded as hub lncRNAs in our prognostic model. GO and KEGG pathway enrichment analyses revealed that MIR205HG was significantly enriched for multiple pathways, including Metabolic pathways (*P* < 0.05) and Ras signaling pathway (*P* < 0.05), as well as GO terms such epidermis development (*P* < 0.001; Figures 4A,B). Moreover, MIAT was significantly enriched for multiple pathways, including Cytokine-cytokine receptor interaction (*P* < 0.001) and Chemokine signaling pathway (*P* < 0.001), as well as GO terms such as immune response (*P* < 0.001; Figures 4C,D). To further explore the correlation between MIAT and immune response, we picked out the genes enriched in Natural killer cell mediated cytotoxicity and T cell receptor signaling pathway. LCK, VAV1, PTPN6, PIK3CG, ZAP70, IFNG, and CD247 were overlapped between the two signaling pathway. Based on the MCP-counter method, we also evaluated the association with MIAT, LCK, VAV1, PTPN6, PIK3CG, ZAP70, IFNG, and CD247 and immune cell populations from transcriptomic data. As shown in Figure 4E and Supplementary Figure 2, the fractions of different immune cells showed significantly positive correlation with the expression of MIAT and its immune related gene. And there was a strong correlation between these genes and T cells, CD8^+^T cells, and NK cells.

## Discussion

Melanoma is known as an aggressive tumor which shows an increasing incidence and poor prognosis in the metastatic phase. The 5-year survival rate is ~90% when the localized disease is detected at an early stage. However, survival rates dramatically decrease to only 5–10% for patients with distant metastasis (28). Thus, identifying novel molecular network biomarkers is needed to stratify patients for earlier detection and to improve the long-term survival rate.

LncRNAs have drawn increasing attention in tumor initiation and progression via its ceRNA activity (29, 30). For instance, Mou et al. (31) confirmed that the oncogenic functions of lncRNA ATB could be attributed to its ceRNA activity to enhance the expression of Yes associated protein 1 by sponging miR-590-5p in malignant melanoma cells. HOTAIR could exert a tumor promoting effect on malignant melanoma and act as a ceRNA suppressing miR-152-3p expression (32). Liang et al. found that lncRNA-ZFAS1 promoted the progression of melanoma through regulation of the miR-150-5p/RAB9A axis (33). LncRNA OIP5-AS1 functioned as a ceRNA participating in glutamine catabolism and melanoma cells growth (34).

In the current study, a total of 2,020 DEmRNAs, 438 DElncRNAs and 65 DEmiRNAs were identified. Based on the melanoma-specific dysregulated RNAs, a ceRNA regulatory network was established, including 39 DElncRNAs, 10 DEmiRNAs, and 16 DEmRNAs. Univariate and multivariate Cox regression analyses were applied to select potential prognosis-related lncRNAs and a 7-lncRNA prognostic signature (MIR205HG, LINC00200, LIFR-AS1, H19, MIAT, AC012640.1, and PLCH1-AS1) was identified. In addition, the potential biological functions of MIR205 and MIAT were also evaluated.

With the goal of identifying lncRNAs significantly associated with OS of melanoma patients, univariate and multivariate Cox proportional regression analyses were carried out. Univariate regression analysis on the DElncRNAs of the ceRNA network identified 15 lncRNAs that were associated with OS. Multivariate analysis showed significant prognostic value of 7 of those lncRNAs in the OS of melanoma patients. A cumulative risk score of the 7 lncRNAs was calculated, which indicated that this 7-lncRNA signature independently predicted OS in melanoma patients. And the AUC value of the ROC curve was all >0.7 when assessing the accuracy of the signature over 3-, 5-, and 10-year OS rates. These results indicate that the 7-lncRNA signature can provide a powerful prognostic tool for patients with melanoma. In addition, there is a well-known repertoire of common driver mutations in melanoma, with the most prevalent being mutations in BRAF, NRAS (26). Patients carrying mutant BRAF or NRAS always have a different prognosis comparing with those carrying wild-type genes. Thus, we reviewed the BRAF or NRAS mutation status in TCGA-SKCM dataset, and our results showed the prognostic values of the 7-lncRNA signature in melanoma patients with different subtypes.

MicroRNA-205 host gene (MIR205HG) has been found as a oncogene in cervical cancer by modulating miR-122-5p/FOXP2 axis (35). In Di et al.'s study (36), they also identified the oncogenic role of lncMIR205HG in head and neck squamous cell carcinoma. However, studies on MIR205HG remain rare, and its biological functions in the control of melanoma tumorigenesis are needed to be characterized. In our study, we identified the predictive ability of MIR205HG based on the ceRNA network, GO and KEGG pathway analyses indicated that the majority of the implicated genes were significantly involved in metabolism and epidermis development-related biological processes mediating tumor progression. Our results showed that MIR205HG might be a potential therapeutic target or candidate prognostic biomarker in melanoma.

Myocardial infarction associated transcript (MIAT) was originally identified as a candidate gene for myocardial infarction, and recently shown to participate in the progression of cancer and the process of metastasis, such as melanoma (37). For instance, Yang et al. reported the high expression of MIAT in melanoma and it promoted cell proliferation, invasion and migration through the PI3K/AKT signaling pathway (37). However, our results indicated that patients with high expression of MIAT carried out a better prognosis. In our study, pathway enrichment analysis suggested that MIAT was significantly enriched in immune response and cytokine-cytokine receptor interaction. Melanoma is characterized by high immunogenicity and is typically infiltrated by different types of immune cells. Our results also showed that the expression of MIAT and its related immune molecules were significantly associated with the infiltration of immune cells, such as T cells, CD8^+^T cells, and NK cells. We speculated that the higher the expression of MIAT, the more sensitive it may be to tumor immunotherapy. Furthermore, the potential strong correlation between MIAT and immune cells suggested the importance of this lncRNA in melanoma, meaning that such a lncRNA could affect prognosis via immune-related pathways.

Another lncRNA LIFR antisense RNA 1 (LIFR-AS1) is targeted by hsa-mir-206, hsa-mir-150, hsa-mir-372, and hsa-mir-373. It was reported that LIFR-AS1 could predict the survival status of patients with clear cell kidney carcinoma. LIFR-AS1 also participated in modulating the resistance of colorectal cancer to photodynamic therapy, which functioned as a ceRNA regulatory network (38). H19 is a long non-coding RNA, and its abnormal expression has been found in various tumors, including melanoma. In current study, H19 is dysregulated between PM and MM. Another study also demonstrated that lncRNA H19 predicts poor prognosis in patients with melanoma and regulates cell growth, invasion, migration, and epithelial-mesenchymal transition (EMT) in melanoma cells (39). And the oncogenic role of H19 was revealed in patients with papillary thyroid carcinoma (40). No study so far has reported any association of LINC00200, AC012640.1, and PLCH1-AS1 with cancer.

As far as we can see, it is the first report integrating a ceRNA network to build a lncRNA-related risk score and evaluate the OS of melanoma patients between PM and MM. This new approach can elucidate the lncRNA-mediated ceRNA regulatory mechanisms in the progression and prognosis of melanoma, and identify novel lncRNAs as potential diagnostic biomarkers or therapeutic targets. Compared to the previous studies (15, 16), our study obtained DElncRNAs between PM and MM rather than stage I–IV. We further explored the relationship between the prognostic model and the common BRAF and NRAS mutant in melanoma.

Although the findings of our study have important clinical implications, the certain limitations must also be noted. First, the prognostic value of 7-lncRNA signature remains to be verified in future studies. Second, previous works have reported some lncRNAs associated with melanoma prognosis, such as DSCAM-AS1, CRNDE, and CACS2, which were not involved in our results. Third, the biological roles of 7 lncRNAs in melanoma also need to be further investigated.

## Conclusion

In summary, we have identified a 7-lncRNA signature as a potential prognostic predictor for melanoma patients by analyzing the genome-wide lncRNA expression data from the TCGA database based on a ceRNA network. The current findings provide novel insights into the lncRNA-related ceRNA network in melanoma and identify potential diagnostic and prognostic biomarkers. Further functional studies are needed to elucidate the molecular mechanisms underlying lncRNA function in melanoma.

## Data Availability Statement

Publicly available datasets were analyzed in this study. This data can be found here: https://cancergenome.nih.gov.

## Author Contributions

NL, ZL, and XL designed the experiments. NL and XL collected the data. NL and ZL analyzed the data. HC, NL, and ZL wrote the manuscript. HC and XL reviewed the manuscript.

### Conflict of Interest

The authors declare that the research was conducted in the absence of any commercial or financial relationships that could be construed as a potential conflict of interest.
