# Supplementary material for: Comprehensive Analysis of a Competing Endogenous RNA Network Identifies Seven-lncRNA Signature as a Prognostic Biomarker for Melanoma
Source: Front Oncol. 2019 Oct 3;9:935. doi: 10.3389/fonc.2019.00935 (PMC6794712; doi:10.3389/fonc.2019.00935)
Supplement: Supplementary file 1 [file Data_Sheet_1.docx]

Supplementary Material

## Supplementary Figures

**
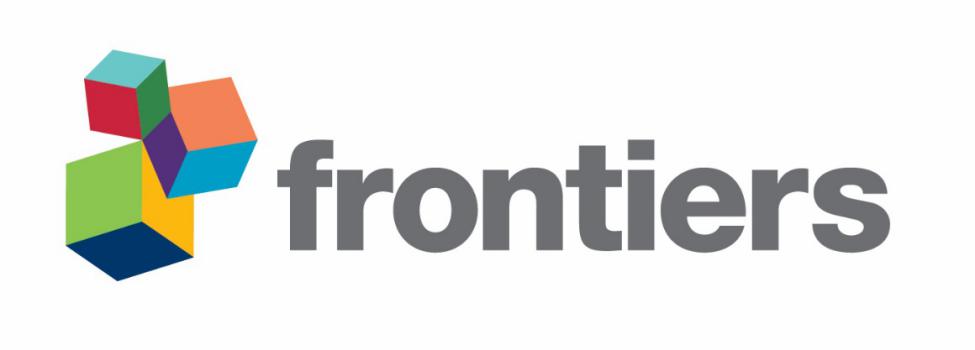
**

**Supplementary Figure 1.** The overlapping mRNAs that were differentially expressed in melanoma patients (left-hand circle) and were coexpressed with lncRNA MIR205HG and MIAT (right-hand circle).


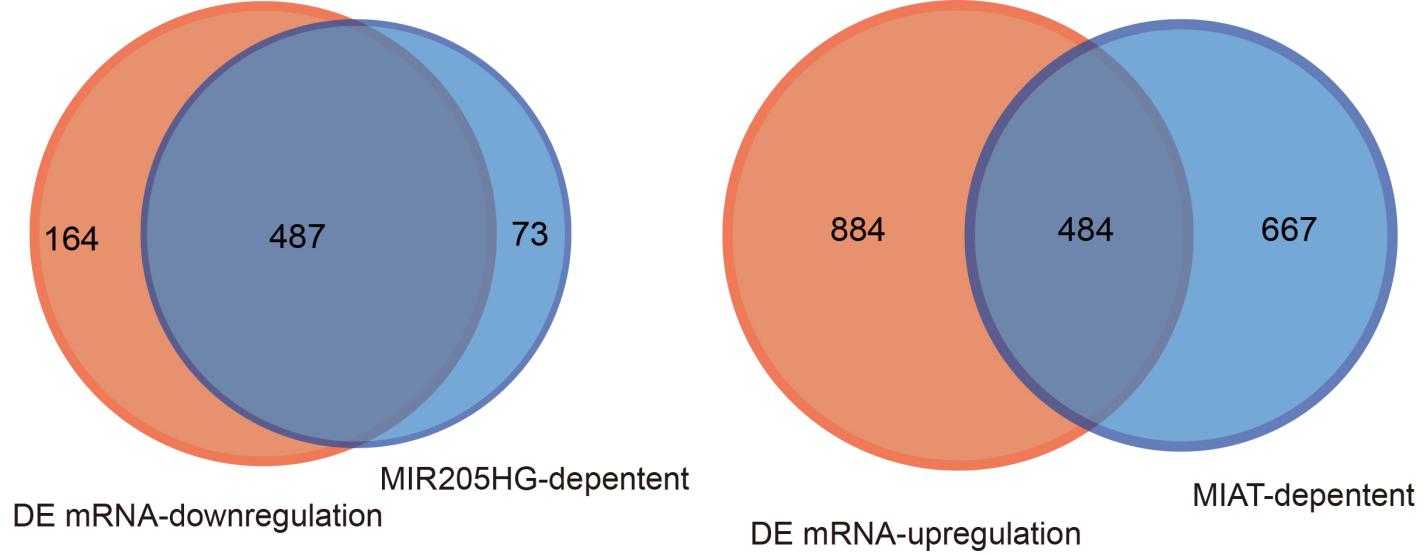


**Supplementary Figure 2.** Correlation of MIAT, MIAT-related immune molecules and immune cell populations. Left: the linear regression plot; right: the Pearson correlation coefficient. Pink: low correlation; red: high correlation.


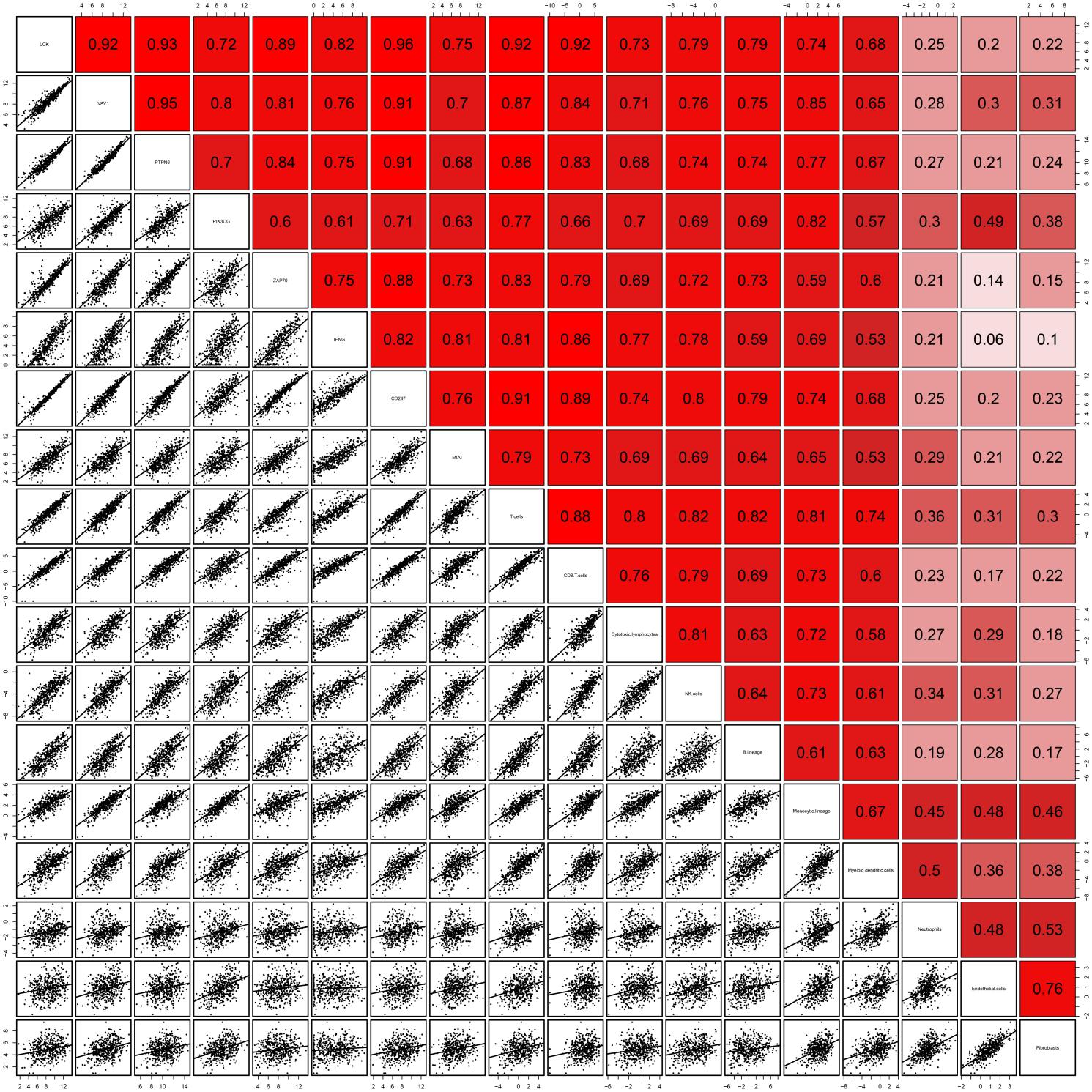


**Supplementary Table 1: 7 prognostic lncRNAs. FC, fold change; FDR, false discovery rate.**

| **LncRNAs** | **Log_2_FC** | **P-value** | **FDR** |
| --- | --- | --- | --- |
| MIR205HG | -4.845135463 | 7.71E-32 | 7.13E-30 |
| LINC00200 | 6.869247868 | 4.86E-16 | 1.83E-14 |
| LIFR-AS1 | 1.636883566 | 8.56E-12 | 1.96E-10 |
| H19 | 1.28991865 | 2.61E-08 | 3.22E-07 |
| MIAT | 1.186827534 | 1.55E-07 | 1.62E-06 |
| AC012640.1 | 1.263689372 | 0.000340661 | 0.001428349 |
| PLCH1-AS1 | 1.720373591 | 0.000599955 | 0.002331341 |
